# Vulvar squamous cell carcinoma due to human papillomavirus type 11

**DOI:** 10.1111/srt.13436

**Published:** 2023-08-11

**Authors:** Pati Aji Achdiat, Retno Hesty Maharani, Nizami Hamada, Reiva Farah Dwiyana, Laila Tsaqilah, Hermin Aminah Usman, Zulfan

**Affiliations:** ^1^ Department of Dermatology and Venereology, Faculty of Medicine Universitas Padjadjaran, Dr. Hasan Sadikin Hospital Bandung West Java Indonesia; ^2^ Department of Anatomical Pathology, Faculty of Medicine Universitas Padjadjaran, Dr. Hasan Sadikin Hospital Bandung West Java Indonesia

Dear Editor,

Vulvar carcinoma is the fourth most common genital malignancy.^1^ One of the most common types of vulvar malignancy is squamous cell carcinoma (SCC).^2^ SCC is a malignancy originating from squamous epithelial cells.^1^ The incidence of SCC of the vulva has increased over the last decade up to 0.6%−5%.^3^ Risk factors for vulvar SCC include advanced age, immunodeficiency, smoking habits, chronic inflammation of the vulva, and human papillomavirus (HPV) infection.^1^, ^2^ HPV is classified into a low‐risk type, which generally causes warts and benign lesions, and a high‐risk type, which generally causes malignant lesions.^4^ However, several studies have reported cases of genital and vulvar malignancies due to low‐risk HPV types.^5^ Here, we describe a rare case of vulvar SCC in a 72‐year‐old woman with positive genotyping polymerase chain reaction (PCR) results for low‐risk HPV type 11.

A 72‐year‐old woman came to the Department of Dermatology and Venereology with main complaint of a yellowish white lump on the genitals that was itchy and painful. The complaint initially felt 1 year before admission in the form of asymptomatic skin‐colored papules that became larger, increased in number, and coalesced until 1 month before admission, became in the form of yellowish white tumors with a verrucous surface, felt itchy and painful. There was no history of sexually transmitted infections or history of risky sexual relations in the patient or the patient's husband. On physical examination, the vital signs were within normal limits, normal weight, and no lymph node enlargement was found. On the venereological status of the vulva, multiple lesions were seen, partially confluent, irregular in shape, measuring 1 × 0.5 × 0.2 cm to 2 × 2.5 × 0.3 cm, well defined, raised, and dry in the form of a yellowish white tumor with a verrucous surface, as shown in Figure 1. The patient was initially diagnosed with suspected vulvar SCC. Routine hematological examination and serologic tests for syphilis, hepatitis B, and HIV were within normal limits. Chest radiography also showed no abnormalities. Biopsy performed on the lesion with histopathological features as shown in Figures 2 and 3 revealed covered epidermis with stratified squamous epithelium, which was partly erosive, ulcerative, and partly became a tumor mass consisting of cells of a round, oval shape, growing hyperplastic, condensed, in groups. Polymorphic, hyperchromatic, and mitotic cell nuclei were found. Invasion of tumor cells into the underlying stroma was seen. The dermis consisted of an edematous fibrocollagenous connective tissue stroma accompanied by inflammatory lymphocytes, polymorphonuclear neutrophils, and histiocytes, especially in the papillary dermis, accompanied by extensive bleeding. The skin adnexa looked normal. Histopathological examination supported the vulvar well‐differentiated SCC. HPV PCR showed positive result for HPV type 11. There were no signs of spread to the lymph nodes or metastases. The final diagnosis was well‐differentiated vulvar SCC FIGO stage I.

**FIGURE 1:**
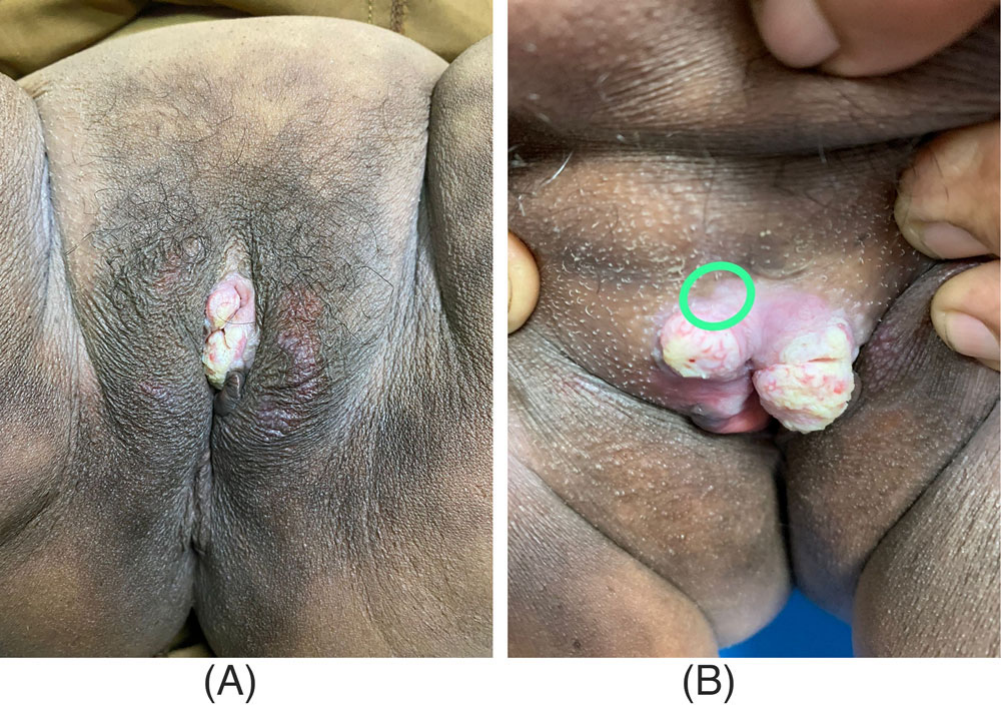
(a and b) Skin lesions on admission day.

**FIGURE 2:**
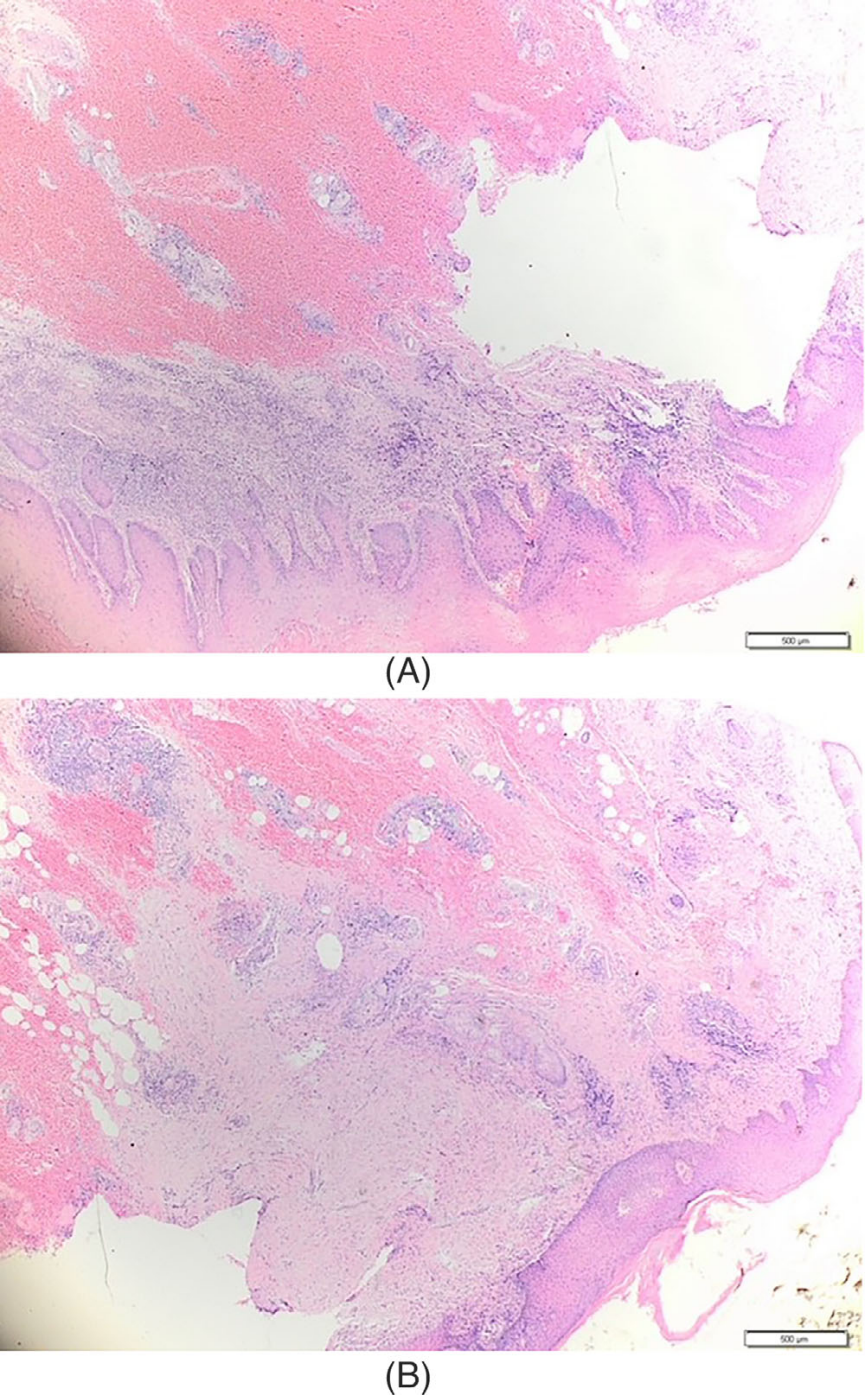
(a and b) The results of histopathological examination at 20× magnification. Stratified squamous epithelium, keratinized, partly ulcerative and partly become a tumor mass.

**FIGURE 3:**
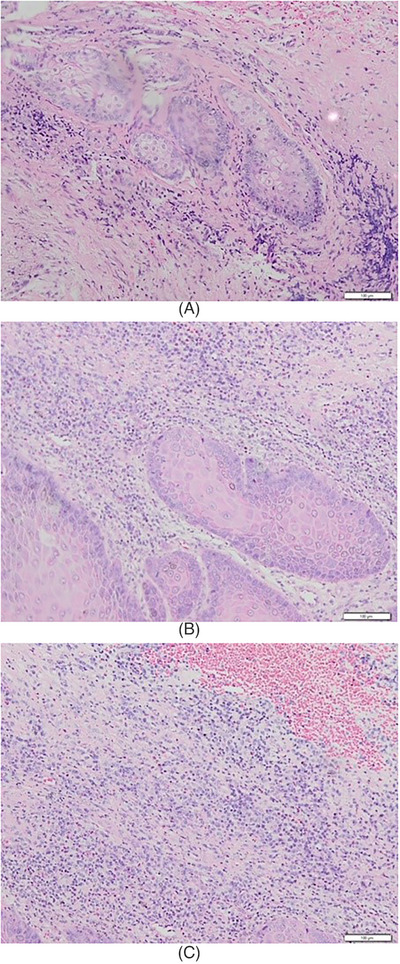
(a–c) The results of histopathological examination at 100× magnification. The tumor mass consisted of cells of a round, oval shape, growing hyperplastic, condensed, in groups. Polymorphic, hyperchromatic, and mitotic cell nuclei were found. Invasion of tumor cells into the underlying stroma was seen.

The prevalence of vulvar SCC is 5% of all cases of genital malignancy in women and has become the most common type of vulvar and vaginal tumor.^2^ The incidence of vulvar malignancy over the last 10 years has increased by 14% in developed countries with increasing age.^3^ Vulvar SCC generally occurs in postmenopausal women with an average age of diagnosis is 70−80 years.^2^ The patient in this case report was a 72‐year‐old woman. The occurrence of HPV infection was significantly linked to having had sexual intercourse for the first time before the age of 16. Moreover, risk factors for vulvar SCC include HPV infection.^6^ In this study, patient had her first coitarche at the age of 15 years.

Based on the nucleotide sequence in the genetic material, there are 150 types of HPV, which are grouped into low‐risk and high‐risk types. Low‐risk HPV types generally cause anogenital warts and benign lesions, while high‐risk HPV types generally cause cancer and malignant lesions. Low‐risk HPV types include types 6, 11, 42, 43, and 44, while high‐risk types include types 16, 18, 31, 33, 35, and 39.^7^ HPV has several proteins that play a role in carcinogenesis, especially early (E)6 and E7 proteins.^1^ E6 and E7 are expressed in HPV‐infected cells. This expression affects different cellular pathways, including deactivation and breakdown of proteins p53 and pRB, leading to uncontrollable cellular growth, proliferation, evasion of tumor suppression, and other characteristics of tumorigenicity.^8^ E6 and E7 affect DNA replication, cell cycle, and modification of host cell immune response.^7^ E6 and E7 proteins in high‐risk HPV types have up to 10 times higher affinity for p53 and Rb proteins compared to low‐risk HPV types.^9^ The expression of high‐risk HPV E6/E7 mRNA was also found to be associated with age. As age increased, the risk of being affected by HPV also increased.^10^ The precursor lesion of the HPV‐dependent SCC vulva is called a high‐grade squamous intraepithelial lesion, which is characterized by the presence of atypical cells due to high‐risk HPV.^1^ However, in a study conducted by Villadsen et al., three cases of vulvar SCC were found with PCR‐positive results for low‐risk HPV types; among others, two cases were caused by HPV type 11 and one case was caused by HPV type 6.^6^ This study reports one case of vulvar SCC due to low‐risk type HPV 11; thus, the risk of malignancy development in even low‐type HPV must be considered, especially in elderly.

## CONFLICT OF INTEREST STATEMENT

The authors declare that there are no conflicts of interest.

## FUNDING INFORMATION

This case report received no specific grant from any funding agency in the public, commercial, or not‐for‐profit sectors.

## ETHICS STATEMENT

Consent for the publication of patient's photographs and medical information was provided by the authors at the time of letter submission to the journal stating that the patient gave consent for her photographs and medical information to be published in print and online and with the understanding that this information may be publicly available. Institutional approval was obtained to publish the case details.
